# *Bacillus velezensis* HN-2: a potent antiviral agent against pepper veinal mottle virus

**DOI:** 10.3389/fpls.2024.1403202

**Published:** 2024-07-10

**Authors:** Zhe Xuan, Yu Wang, Yuying Shen, Xiao Pan, Jiatong Wang, Wenbo Liu, Weiguo Miao, Pengfei Jin

**Affiliations:** ^1^ College of Plant Protection, Hainan University/Key Laboratory of Green Prevention and Control of Tropical Plant Diseases and Pests (Hainan University), Ministry of Education, Haikou, China; ^2^ School of Life and Health Sciences, Hainan University, Haikou, China

**Keywords:** biocontrol agent, PVMV, antiviral, induced systemic resistance, plant immune, *Bacillus velezensis*, *Potyvirus*, pepper veinal mottle virus

## Abstract

**Background:**

Pepper veinal mottle virus (PVMV) belongs to the genus *Potyvirus* within the family Potyviridae and is a major threat to pepper production, causing reduction in yield and fruit quality; however, efficient pesticides and chemical treatments for plant protection against viral infections are lacking. Hence, there is a critical need to discover highly active and environment-friendly antiviral agents derived from natural sources. *Bacillus* spp. are widely utilized as biocontrol agents to manage fungal, bacterial, and viral plant diseases. Particularly, *Bacillus velezensis* HN-2 exhibits a strong antibiotic activity against plant pathogens and can also induce plant resistance.

**Methods:**

The experimental subjects employed in this study were *Bacillus velezensis* HN-2, benzothiadiazole, and dufulin, aiming to evaluate their impact on antioxidant activity, levels of reactive oxygen species, activity of defense enzymes, and expression of defense-related genes in Nicotiana benthamiana. Furthermore, the colonization ability of *Bacillus velezensis* HN-2 in Capsicum chinense was investigated.

**Results:**

The results of bioassays revealed the robust colonization capability of *Bacillus velezensis* HN-2, particularly in intercellular spaces, leading to delayed infection and enhanced protection against PVMV through multiple plant defense mechanisms, thereby promoting plant growth. Furthermore, *Bacillus velezensis* HN-2 increased the activities of antioxidant enzymes, thereby mitigating the PVMV-induced ROS production in *Nicotiana benthamiana*. Moreover, the application of *Bacillus velezensis* HN-2 at 5 dpi significantly increased the expression of JA-responsive genes, whereas the expression of salicylic acid-responsive genes remained unchanged, implying the activation of the JA signaling pathway as a crucial mechanism underlying *Bacillus velezensis* HN-2-induced anti-PVMV activity. Immunoblot analysis revealed that HN-2 treatment delayed PVMV infection at 15 dpi, further highlighting its role in inducing plant resistance and promoting growth and development.

**Conclusions:**

These findings underscore the potential of *Bacillus velezensis* HN-2 for field application in managing viral plant diseases effectively.

## 1 Introduction

The pepper cultivar *Capsicum chinense* is a local germplasm of the domesticated species, cultivated exclusively in Hainan Island, China (Hu et al., 2020). *C. chinense* is widely utilized for preparing canned sauce, a favorite commercial product among tourists. The increased production of *C. chinense* in Hainan has led to numerous plant disease outbreaks. Notably, five plant viral diseases, including the chilli veinal mottle virus (ChiVMV, *Potyvirus*), pepper veinal mottle virus (PVMV, *Potyvirus*), chilli ringspot virus (ChiRSV, *Potyvirus*), tobacco mosaic virus (TMV, *Tobamovirus*), and cucumber mosaic virus (CMV, *Cucumovirus*), are prevalent in the Hainan province fields, causing considerable economic losses over the last decade. PVMV was initially identified in Eastern Ghana in 1971 (Brunt and Kenten, 1971) and belongs to the genus *Potyvirus*, family Potyviridae, sharing characteristics with other potyviruses (Alegbejo and Abo, 2002; Ha et al., 2008; Matsumoto et al., 2016). PVMV infects a variety of hosts, such as pepper, tomato, tobacco, eggplant, petunia, *Solanum nigrum* L., *S. integrifolim* Poir., *Datura metel*, *D. stramonium*, *Physalis angulata*, and *P. micrantha* (Brunt et al., 1978; Givord, 1982; Atiri and Ligan, 1986; Alegbejo, 1999). Liang et al. (2015) reported a PVMV infection incidence of 74.07% in Wenchang and Wanning, Hainan, which significantly impacts pepper production and fruit quality (Fajinmi and Odebode, 2010). Despite its prevalence, effective pesticides and chemical treatments against PVMV are limited, necessitating the development of novel antiviral agents and resistant varieties.

Currently, effectively controlling the virus once a plant has been infected remains a challenge for farmers. The primary methods for virus control involve cultivating resistant varieties, developing and utilizing natural products, and using chemicals and synthetic compounds. The potential concept of chemically mediated plant virus control relies on compounds that activate the plant immune system. When locally infected with a necrotizing pathogen or non-pathogen, plants often develop long-lasting, broad-spectrum “immunity” against subsequent infection (Kunz et al., 1997). Benzo(1,2,3)-thiadiazole-7-carbothioic acid S-methyl ester (BTH) is a synthetic compound capable of inducing disease resistance in several dicotyledonous and monocotyledonous plant species (Friedrich et al., 1996). Friedrich et al. (1996) found that BTH cannot cause the accumulation of salicylic acid (SA) but can induce disease resistance and *nahG* gene expression, thus activating the SAR signal transduction pathway at the site or downstream of SA accumulation. These results demonstrate the disease control mechanism of BTH by which it activates SAR. Dufulin is a novel antiviral agent that is highly effective against plant viruses and is widely used to prevent and control viral diseases in tobacco and rice in China (Song et al., 2009). Chen et al. (2012) found that HrBP1 is a target protein of dufulin, which can also activate the SA signaling pathway to induce host plants to generate antiviral responses.

However, in recent decades, plant growth-promoting rhizobacteria (PGPRs) have been shown to interact symbiotically and synergistically and effectively colonize the rhizosphere (Ongena and Jacques, 2008). They represent a mutually helpful plant–microbe interaction. Plant growth is enhanced by PGPR through antibiosis, the induction of systemic resistance, and competitive multiplication. The most crucial biocontrol trait of PGPR is their ability to trigger an immune reaction in plant tissues, leading to a systemically expressed resistance state that renders the host less susceptible to subsequent infection (induced systemic resistance, ISR) (Mariuotto and Ongena, 2015; Pršić and Ongena, 2020). PGPR can be beneficial to plants and can perform the same function as chemical fertilizers, pesticides, and elicitors do. *Bacillus* is an important and well-characterized model organism of plant growth-promoting rhizobacteria. *Bacillus subtilis* belongs to a new class of MAMPs. It can effectively inhibit plant activity and ISR, which act as elicitors of plant immunity (Boutrot and Zipfel, 2017; Radhakrishnan et al., 2017). *Bacillus* also produces various metabolites such as lipopeptides, hydrolytic enzymes, and bacterial volatile compounds (BVCs) (Cazorla et al., 2007; Wang et al., 2018; Rajaofera et al., 2019; Jin et al., 2020a, b). Notably, *Bacillus* spp. synthesize antibiotic lipopeptides, including surfactin, iturin, and fengycin (Hashem et al., 2019). Fengycin and surfactin can interact with plant cells as bacterial determinants to turn on an immune response through the stimulation of the induced systemic resistance phenomenon. García-Gutiérrez et al. (2013) showed that ISR is activated by *Bacillus* spp. through the induction of the synthesis of jasmonic acid, ethylene, and *NPR1* regulatory genes in plants. Some studies have indicated that *Bacillus* spp. produce secondary metabolites, such as lipopeptides, which can stimulate and initiate the activities of key enzymes of the oxylipin pathway in tomatoes (Blée, 2002). The phenolic or phenylpropanoid metabolic pathway is also well known to be stimulated concomitantly by the activation of plant defense reactions (Dixon et al., 2002). Ongena et al. (2005) found that when potato tuber cells were treated with fengycins produced by *Bacillus*, the accumulation of plant phenolics was involved in or derived from phenylpropanoid metabolism. Jayaraj et al. (2004) reported that phenylalanine ammonia-lyase, peroxidase, and *de novo* protein synthesis in plants was activated; these enzymes were produced when *Bacillus* was applied to plants. Furthermore, *Bacillus* and BVCs exhibit potent antiviral activities against various plant viruses, including cucumber mosaic cucumovirus (CMV), tomato mottle virus (TMV), pepper mottle virus (PepMoV), tomato yellow leaf curl virus (TYLCV), and tomato spotted wilt virus (TSWV) (Ongena and Jacques, 2008; Kong et al., 2018). In addition, Wu et al. (2017) found that *B. amyloliquefaciens* FZB42 strains produce two types of cyclodipeptides that can induce resistance against TMV infection in *N. benthamiana* by activating the SA-mediated plant defense pathway. *Bacillus* spp. are promising candidates for broad-spectrum antiviral therapy (Ongena and Jacques, 2008). However, despite significant advances made over the past few decades in understanding the regulation of hormonal modulation by PGPR and the induction of acquired systemic resistance in plants (Pieterse et al., 2012, 2014; Köhl et al., 2019), we are still far from having a clear picture of the intricate immune-related molecular events and resistance pathways induced by biocontrol microorganisms.

In a previous study, the cyclic lipopeptide C_15_ surfactin, isolated from *Bacillus velezensis* HN-2, was shown to induce systemic resistance to pathogens in plants, thereby contributing to their biocontrol activity. Here we present a novel approach wherein *Bacillus velezensis* HN-2 is capable of eliciting an immune response in plant tissues, resulting in systemic resistance against PVMV and exhibiting strong colonization. This suggests potential for future field applications in managing plant viral diseases.

## 2 Materials and methods

### 2.1 Bacterial strains’ culture conditions and chemical compounds

*B. velezensis* strain HN-2, which was stored at CCTCC (ID.CCTCC M 2018382) and cultured in lysogeny broth (LB) medium, was centrifuged at 200 rpm for 48 h at 28°C to separate bacterial cells, remove supernatants, and collect cultures, which were adjust to the desired optical density at 600 nm (OD600 = 0.9) and irrigated on soil and plant root. *B. velezensis* strain HN-2-GFP was cultured in chloramphenicol (5 μg mL^-1^) LB medium. Then, 50 mg mL^-1^ benzothiadiazole and dufulin solution was smeared on whole leaves. Benzothiadiazole (99% purity; Aladdin Co., China) and dufulin (99% purity; Tianyuan Co., China) were used as positive controls, and sterile water (CK) was used as the blank control, respectively.

### 2.2 Plant materials

Tobacco seeds (*Nicotiana benthamiana L.*) were surface-sterilized for 3 min in 75% ethanol, rinsed with sterile water for five times, and then germinated in 1/2 MS medium in a growth chamber maintained at 25°C (24 h in the dark). Following germination, the seedlings were transferred as plantlets, filled with autoclaved soil consisting of 1:1 (v/v) high-nutrient soil and vermiculite in pots, and then cultured in a growth chamber at 25°C/25°C (14-h light/10-h dark) with 70% relative humidity and observed daily for symptom development recording [Pepper (*capsicum chinense*): Cooperative 903 Big Red Pepper (Shanghai Pepper Institute)].

### 2.3 Agroinfiltration

Similarly, *N. benthamiana* plants at the seventh leaf stage were carefully selected and subjected to infiltration with *Agrobacterium tumefaciens* (GV3101 + PVMV) cultures containing the relevant plasmids. The cultures were adjusted to the desired optical density at 600 nm (final OD600 = 1) and infiltrated into the leaf tissues of *N. benthamiana* plants essentially (Cui and Wang, 2017). The leaves were inoculated with the virus after 48 h and cultivated in a greenhouse. Five treatments were adopted: sterile water, sterile water + pHNu-GFP, sterile water + benzothiadiazole + pHNu-GFP, sterile water + dufulin + pHNu-GFP, and sterile water + HN-2 + pHNu-GFP, respectively.

### 2.4 Diaminobenzidine staining and enzyme activity determination

Superoxide dismutase (SOD), peroxidase (POD), catalase (CAT), and malondialdehyde (MDA) activities were determined according to the protocol provided by the manufacturer (Nanjing Jiancheng Biology Institution, Nanjing, China). The diaminobenzidine (DAB) (BBI Life Sciences, China) test was used to detect reactive oxygen species (ROS) production, and the method is as described previously (Liu et al., 2020). Tissue samples were collected at 1, 3, 5, 7, and 15 days after the inoculation treatment for assays on defensive enzyme activities and observation by DAB staining. All of the measurements were performed in triplicate.

### 2.5 Phytohormone content measurements

The concentration of salicylic acid (SA) was determined using a previously established method with minor modifications (Nakano and Mukaihara, 2018). Tissue samples were collected at 1, 3, 5, 7, and 15 days after inoculation treatment and extracted twice at 4°C for 1 h using 400 µL of an extraction solvent (10% methanol and 1% acetic acid). The standard salicylic acid was purchased from Sigma. A subsequent analysis was conducted using high-performance liquid chromatography on an Agilent 1260 Infinity II LC system equipped with a C18 column. Chromatographic separation utilized solvent A (0.1% formic acid in water) and solvent B (0.1% formic acid in acetonitrile) following a gradient elution: starting at 10% solvent B for 1 min, 95% solvent B for 10 min, maintaining at this level for 2 min, returning to the initial conditions with 10% solvent B for 0.1 min, and finally equilibrating for 2 min at 10% solvent B condition with a constant flow rate of 0.3 mL min^-1^.

### 2.6 RNA extraction and RT-PCR

Tissue samples were collected at 1, 3, 5, 7, and 15 days after inoculation treatment and frozen in liquid nitrogen for subsequent RNA isolation. The quality of extracted RNA should be given following the RNAprep pure KIT (Tiangen), and complementary DNA (cDNA) was synthesized with the Prime Script RT-PCR kit (TaKaRa, Japan). For the qRT-PCR assay, each biological treatment was carried out in three replicates using the SYBR Premix EX Taq kit (TaKaRa) in 20 μL on the ABI Prism 7500 system. The program consisted of a HotStart activation step at 95°C for 14 s, followed by 40 cycles of 95°C for 15 s, 59°C for 30 s, and 72°C for 30 s. The 2^-△△CT^ method (Livak and Schmittgen, 2001) was used to precisely quantify and calculate the relative transcriptional level of each tested gene. All of the measurements were performed in triplicate. The expressions of eight target genes (*Cat1*, *Rboh*, *PAL*, *Ja*, *PR-1b*, *PR3*, *PR5*, and *NPR1* gene) were monitored with qRT-PCR. The *ACT1* gene was used as the internal reference. The gene primers are listed in **Supplementary Table S1**.

### 2.7 Western blotting analysis

Total protein collection and western blotting analysis were performed using a previously reported method (Cui and Wang, 2016). The total proteins were extracted from fresh leaf tissues of *N. benthamiana* plants which were collected at 1, 3, 5, and 15 days after inoculation treatment, and fresh plant leaves were ground to a fine powder in liquid nitrogen. Briefly, protein samples were subjected to electrophoresis on 12% sodium dodecylsulfate–polyacrylamide gel electrophoresis (SDS-PAGE) and electroblotting onto a polyvinylidene difluoride membrane (Immobilon), followed by Western blotting assays using anti-GFP antibodies. Goat anti-rabbit immunoglobulin antibody (Abcam) conjugated to horseradish peroxidase was used as the secondary antibody. The hybridization signals in the blotted membranes were detected with the substrates of enhanced chemiluminescence detection reagents (Thermo Fisher Scientific) and visualized under an ImageQuant LAS 4000 mini biomolecular imager (GE Healthcare).

### 2.8 *B. velezensis* HN-2 colonization assay in *Capsicum chinense*

Identical *Capsicum chinense* seeds were selected for the colonization assay. The colonization assay was performed using a previously reported method (Deng et al., 2019). The plants were kept in square pots (5 × 5 cm), at one plant per pot with nutrition soil, in a greenhouse at a consistent temperature of 30°C. Then, 10 mL of sterile water containing 10^6^ colony-forming units (CFUs) g^-1^ of *B. velezensis* strain HN-2-GFP with Cm (chloramphenicol) label was inoculated into the soil of 1-month generated *C. chinense* seedlings. A total of 300 plants were set for the treatment. After inoculation for 3, 5, 15, 30, and 45 days, nine plants were harvested each time for investigation of *B. velezensis* strain HN-2-GFP colonization. The collected roots with rhizosphere soil were put into 50-mL tubes with 10 mL of sterile water and sonicated three times at 30 s cycle^-1^. The washing buffer during the three times was subjected to low-speed centrifugation (1,000 × *g*, 10 min). The pellets were set as a rhizosphere soil sample. The roots were transferred into a new tube containing 10 mL of sterile water. The roots were sonicated for another three rounds of 30 s cycle^−1^. After the roots were put into another 10-mL tube with fresh sterile water, the roots were ground using an electric pestle. The homogenate was set as root sample. The samples were used to spread the plates with 5 μg mL^-1^ chloramphenicol after properly diluting. CFUs were calculated. The CFU of the rhizosphere soil samples and roots was normalized by using the corresponding root fresh weight with rhizosphere soil from each plant.

### 2.9 Antivirus tests in the field

To test the protective effect of *B. velezensis* HN-2 on plant protection and antiviral function, after 60 days of growth, a total of 300 *Capsicum chinense* with the same growth trend were transplanted in the field. The strain HN-2 (OD600 = 0.9) was irrigated on the soil of *C. chinense*. Benzothiadiazole, dufulin, and sterile water were sprayed on the leaves of *C. chinense*. The sterile water served as a blank control, and the plants that were inoculated with PVMV-GFP is the negative control. Benzothiadiazole and dufulin were used as positive controls. At 2 days after spraying or irrigation, the plants were inoculated with PVMV. The accumulation of PVMV-GFP protein was detected by Western blotting at 60 days post-inoculation (dpi). The infection of PVMV-GFP in *C. chinense* was observed at 180 dpi. Five samples per treatment were collected for further virus detection. The above-mentioned experiments were repeated five times, with nine plants each time.

### 2.10 Statistical analyses

All experiments and data presented here involved at least three repeats. The statistical analyses were all performed using SPSS (version 19.0; SPSS Inc). All data were expressed as means ± SD. Different letters indicate statistically significant differences between treatments according to Duncan’s multiple-range test at *P <*0.05, and the independent-samples test was used to test the significance of the difference.

## 3 Results

### 3.1 Effect of *B. velezensis* HN-2 in defense against PVMV

**Figure 1:**
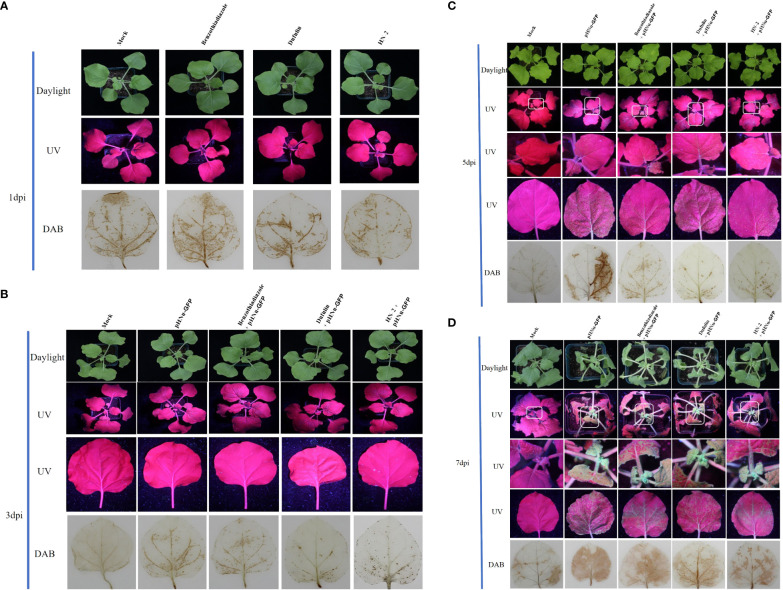
Persistent antiviral effect of benzothiadiazole, dufulin, and *B*. *velezensis* HN-2 on *N. benthamiana*; each treatment group was inoculated with PVMV-GFP on 3, 5, and 7 days, respectively. The leaves of *N. benthamiana* were treated with sterile water, benzothiadiazole, and dufulin at a concentration of 50 mg mL^-1^, while *B*. *velezensi*s strain HN-2-GFP irrigation on soil and plant root was at a concentration of 5 μg mL^-1^. After 48 h of pretreatment, *N. benthamiana* plants at the seventh leaf stage with a similar growth were infiltrated with *Agrobacterium tumefaciens* (GV3101 + PVMV) cultures containing the relevant plasmid and cultivated in a greenhouse environment. DAB staining was used to detect the production of H_2_O_2_ on *N. benthamiana*. **(A)** Green GFP fluorescence signals (under UV light) and DAB staining were observed after benzothiadiazole, dufulin, HN-2, and sterile water treatments for 1 dpi (that is, representing 24 h of pretreatment). **(B)** Green GFP fluorescence signals (under UV light) and DAB staining were observed after benzothiadiazole, dufulin, HN-2, and sterile water treatments for 3 dpi and friction inoculation of PVMV-GFP at 1 dpi. **(C)** Green GFP fluorescence signals (under UV light) and DAB staining were observed after benzothiadiazole, dufulin, HN-2, and sterile water treatments for 5 dpi and friction inoculation of PVMV-GFP at 3 dpi. **(D)** Green GFP fluorescence signals (under UV light) and DAB staining were observed after benzothiadiazole, dufulin, HN-2, and sterile water treatments for 7 dpi and friction inoculation of PVMV-GFP at 5 dpi. “Mock” means sterile water treatment.

**Figure 2:**
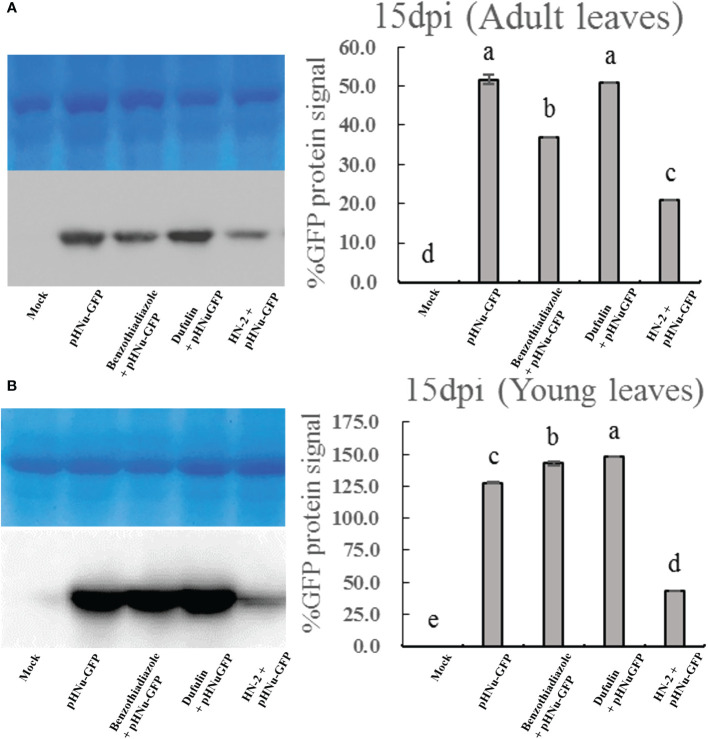
Western blotting analysis showing the level of the PVMV-GFP protein in the adult leaves **(A)** and young leaves **(B)** of *N. benthamiana* at 15 dpi, which were treated with sterile water (mock), pHNu-GFP, benzothiadiazole-, dufulin-, and *B*. *velezensis* HN-2-pHNu-GFP. Values represent means standard deviations (SDs) from three independent experiments; different lowercase letters indicate a highly significant difference (P < 0.05).

To evaluate the resistance of *B. velezensis* HN-2 against PVMV, sterile water (CK) served as the blank control, and pHNu-GFP was used as the negative control. Benzothiadiazole and dufulin were used as positive controls. We pretreated *N. benthamiana* leaves with water, benzothiadiazole, dufulin, and *B. velezensis* HN-2 (**Figure 1A**). Subsequently, pHNu-GFP was inoculated through *Agrobacterium* infection (agroinfiltration) at 48 h post-pretreatment. The subsequent infection of pHNu-GFP was visualized at 1, 3, and 5 dpi (that is, at 3, 5, and 7 dpi of the experimental treatments) under UV light and DAB staining. As shown in **Figures 1A, B**, no damage was observed and no green fluorescence was detected in *N. benthamiana* leaves at 1 and 3 dpi. **Figures 1C, D** show that the GFP fluorescent signals visualized in the inoculated leaves treated with HN-2 were much lower than those of the negative control and positive controls at 5 and 7 dpi. At 7 dpi, GFP fluorescence was detected in the young leaves of the positive and negative plants due to the systemic movement of PVMV-GFP. However, in the young leaves of HN-2-treated plants, slightly expanded GFP signals were visualized at 7 dpi (**Figure 1D**). In addition, we measured the accumulation of PVMV-GFP protein by using Western blotting in the inoculated leaves at 15 dpi. The expression level of PVMV-GFP in both adult and young leaves of the plants treated with *B. velezensis* HN-2 was pronouncedly lower than that of the other treatment groups, with a particularly pronounced effect observed in young leaves (**Figures 2A, B**). This is in line with the fluorescence signals observed under UV light. Furthermore, in comparison to therapeutic efficacy against PVMV achieved by treatments involving benzothiadiazole and dufulin, the results strongly indicated that *B. velezensis* HN-2 exhibits a significant therapeutic effect on *N. benthamiana* plants following PVMV infection.

### 3.2 *B. velezensis* HN-2 alleviates PVMV-induced oxidative damage

DAB is oxidized by hydrogen peroxide in the presence of some haem-containing proteins, which was performed to generate a dark brown precipitate. This precipitate is used as a stain to detect the presence and distribution of hydrogen peroxide in plant cells (Daudi and O’Brien, 2012). Upon treatment with benzothiadiazole, dufulin, and *B. velezensis* HN-2, which can induce plants to produce peroxide, black precipitates were produced after staining using DAB. To observe the continuous induction of ROS by five treatments, the accumulation of H_2_O_2_ was detected in the leaves at 1, 3, 5, and 7 dpi after spraying for 2 days (**Figure 1**). The results revealed the appearance of dark brown precipitates along the leaf veins across all treatment groups. **Figure 1A** demonstrates that uninfected *N. benthamiana* plants did not exhibit the spontaneous formation of dark brown precipitates. From the first day after PVMV inoculation, that is, at 3 dpi, an increasing number of dark spots was observed exclusively in pHNu-GFP-treated plants due to the combined effects of PVMV infection and pharmaceutical treatments. There was an increase in benzothiadiazole- and dufulin-treated plants, but they were significantly different from plants which were only treated with pHNu-GFP. Additionally, *B. velezensis* HN-2 treatment resulted in a significantly fewer black spots than the other treatments. The dark brown precipitates exhibited similar patterns between benzothiadiazole-treated *N. benthamiana* leaves inoculated with pHNu-GFP at 3, 5, and 7 dpi compared to dufulin-treated plants (**Figures 1B–D**), where at 7 dpi they accounted for approximately two-thirds of the leaf area covered by precipitation. Furthermore, as treatment time increased substantially, so did the amount of dark brown precipitates; notably at 7 dpi, they were detected throughout *N. benthamiana* leaves treated only with pHNu-GFP. **Figure 1D** illustrates that adult leaves treated with HN-2 showed less accumulation of dark brown precipitates than young leaves as indicated by decreased staining at 5 dpi, suggesting that HN-2 can reduce the oxidative damage in leaves caused by PVMV. The oxidative damage effects of the plants were significantly weaker than those of BTH and dufulin treatments.

### 3.3 Determination of defense enzyme activities and SA accumulation

**Figure 3:**
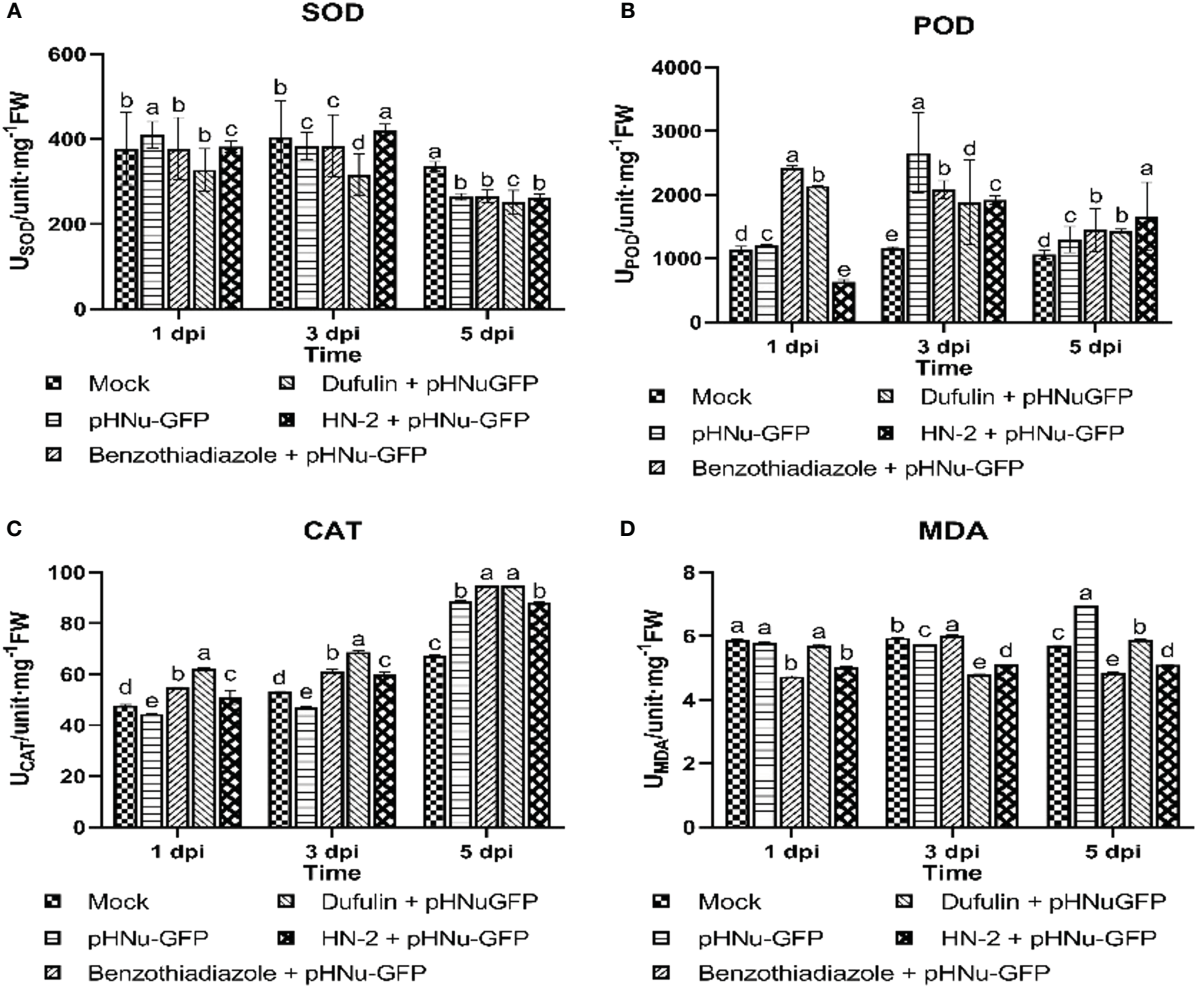
Antioxidant-related enzyme activaties in *N. benthamiana.* Tissue samples were collected after sterile water (mock), pHNu-GFP, benzothiadiazole-, dufulin-, and *B*. *velezensis* HN-2-pHNu-GFP treatments at 1, 3, and 5 dpi (that is, cultures were incubated for 3, 5, and 7 dpi) for assays on defensive enzyme activity assays. **(A)** Superoxide dismutase (SOD), **(B)** peroxidase (POD), **(C)** catalase (CAT), and **(D)** malondialdehyde (MDA). All experiments were repeated three times, and similar results were obtained. Data are presented as the means ± SD from three independent experiments. Values represent means standard deviations (SDs) from three independent experiments; different lowercase letters indicate a highly significant difference (P < 0.05).

Due to the crucial role of antioxidant enzymes in plant defense mechanisms against various plant pathogens, the current work aimed to evaluate the activities of antioxidant enzymes, including superoxide dismutase, peroxidase, catalase, and malondialdehyde. The objective was to determine whether there were induced antioxidant activities after inoculation with pHNu-GFP in the five treatments, which were clearly differentiated by HN-2 treatments (**Figure 3**). SOD enzymes play a major role in detoxifying reactive superoxide (O^2-^) species into H_2_O_2_, which is subsequently degraded by catalases (El-Gendi et al., 2022). As depicted in **Figure 3A**, the results indicated a slight enhancement of approximately 1.07-fold (409.85 unit mg^-1^ FW) in SOD activity following PVMV treatment, which was not significantly different from the positive controls and HN-2-treated plants after PVMV-GFP inoculation compared to the blank control at 1 dpi. This observation could be attributed to an initial response of the plant defense system to oxidative stress. However, at 3 dpi, HN-2-treated plants exhibited the highest SOD activity with a peak value of 421.13 unit mg-1 FW, representing increases of 1.10-, 1.33-, and 1.04-fold when compared to BTH-treated, dufulin-treated, and blank control samples, respectively. Subsequently, there was a decline in SOD activity at 5 dpi.

Antioxidant enzymes, such as peroxidase, play a pivotal role in the conversion of H_2_O_2_ to H_2_O (Gill and Tuteja, 2010). The results of the enzyme activity assay revealed a significant reduction in POD activities by 80.09% in the HN-2 treatment plants (635.62 unit mg^-1^ FW) compared to the blank control (1,144.68 unit mg^-1^ FW) at 1 dpi. Following inoculation of *N. benthamiana* with pHNu-GFP for 3 days, the highest POD activity was observed in the PVMV treatment (2,655.43 unit mg^-1^ FW), followed by BTH treatment (2,078.14 unit mg^-1^ FW), HN-2 treatment (1,916.74 unit mg^-1^ FW), and dufulin treatment (1,884.14 unit mg^-1^ FW) when compared to the blank control (**Figure 3B**). Furthermore, contrasting patterns were observed in changes of POD activity at 5 dpi; the HN-2 treatment demonstrated the highest level at 1,660.31 unit mg^-1^ FW, indicating a 1.15-, 1.16-, and 1.57-fold increase in activity compared to the BTH treatment (1,447.06 unit mg^-1^ FW), dufulin treatment (1,430.72 unit mg^-1^ FW), and blank control (1,060.72 unit mg^-1^ FW), respectively.

The activity of the antioxidant enzyme CAT gradually increased in all treatments and reached its peak at 5 dpi. Remarkably, the BTH-treated and dufulin-treated groups exhibited significantly higher levels of CAT content, with a significant increase of 1.07- and 1.08-fold compared to HN-2 treatments, respectively. Additionally, there was a slight but insignificant increase in CAT activity values exhibited in HN-2 treatments (50.79 and 60.00 unit mg^-1^ FW, respectively) by 1.06- and 1.13-fold compared to the blank control (47.75and 53.24 unit mg^-1^ FW, respectively) at 1 and 3 dpi. Meanwhile, it increased by 1.31-fold compared with the blank control at 5 dpi (**Figure 3C**).

Malondialdehyde (MDA), as a marker for oxidative stress, could be a great indicator of membrane disruption in plants attacked by pathogens (Loreto and Velikova, 2001; Lv et al., 2020). Interestingly, we found that MDA activity remained relatively stable with prolonged HN-2 treatment (5.03 unit mg^-1^ FW, 5.12 unit mg^-1^ FW, and 5.10 unit mg^-1^ FW, respectively), exhibiting a significant reduction in MDA content compared to PVMV treatment (5.80 unit mg^-1^ FW, 5.74 unit mg^-1^ FW, and 6.94 unit mg^-1^ FW, respectively) and the blank control (5.87 unit mg^-1^ FW, 5.94 unit mg^-1^ FW, and 5.69 unit mg^-1^ FW, respectively), representing decreases of approximately 13.28%, 10.80%, and 26.60% and reductions of approximately 14.31%, 13.80%, and 10.37%, respectively. In addition, HN-2 treatments on *N. benthamiana* resulted in a considerable reduction in MDA content when compared to both PVMV treatment and blank control. Furthermore, BTH treatments (4.73 unit mg^-1^ FW at 1 dpi and 4.86 unit mg^-1^ FW at 5 dpi) and dufulin treatment (4.80 unit mg^-1^ FW at 3 dpi) showed a minimal buildup of MDA activity compared to HN-2 treatments, respectively. On the contrary, dufulin treatment exhibited an increase of approximately 1.17-fold at 3 dpi, while dufulin treatment showed an increase of 1.13- and 1.16-fold at 1 and 5 dpi, respectively, in comparison with HN-2 treatment (**Figure 3D**).

**Figure 4:**
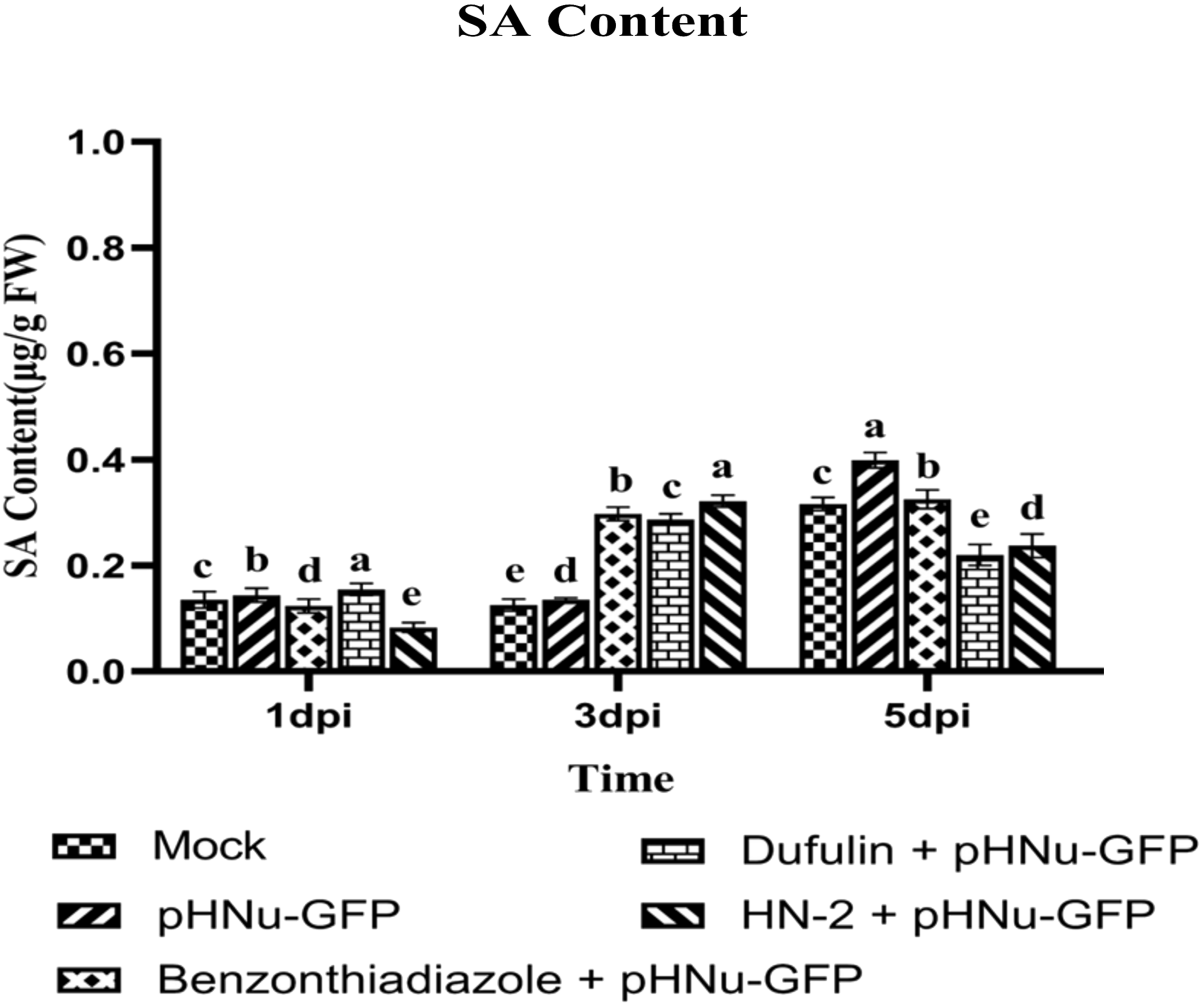
Persistent SA activation effects of sterile water (mock), pHNu-GFP, benzothiadiazole-, dufulin-, and HN-2-pHNu-GFP on *N. benthamiana*. PVMV-GFP in the inoculated leaves of *N. benthamiana* at 1, 3, and 5 dpi at the same time—that is, cultures were incubated for 3, 5, and 7 dpi. Data are presented as the means ± SD from three independent experiments. Values represent means standard deviations (SDs) from three independent experiments; different lowercase letters indicate a highly significant difference (P < 0.05).

Salicylic acid (SA), a crucial signaling molecule involved in plant disease resistance and playing a central role in orchestrating induced plant defense by activating several defense-related genes, leading to SAR-induced signal transduction in plants, was quantified using high-performance liquid chromatography (HPLC). In *N. benthamiana* leaves inoculated with HN-2, the SA content gradually increased and peaked at 3dpi, reaching the highest levels of 0.322 μg g^-1^ FW among five treatments, which represented a slight enhancement of approximately 1.08- and 1.12-fold when compared to BTH-treated (0.297μg g^-1^ FW) and dufulin-treated (0.287μg g^-1^ FW). Moreover, there was no significant difference between the SA content in HN-2 treatment (0.237 μg g^-1^ FW) and those treated with dufulin (0.220 μg g^-1^ FW), but compared to BTH treatment and PVMV treatment and blank control, decreases of 26.97%, 40.52%, and 24.93%, respectively, were shown (**Figure 4**).

### 3.4 Effect of HN-2 on the expression of defense-related genes and jasmonic acid

**Figure 5:**
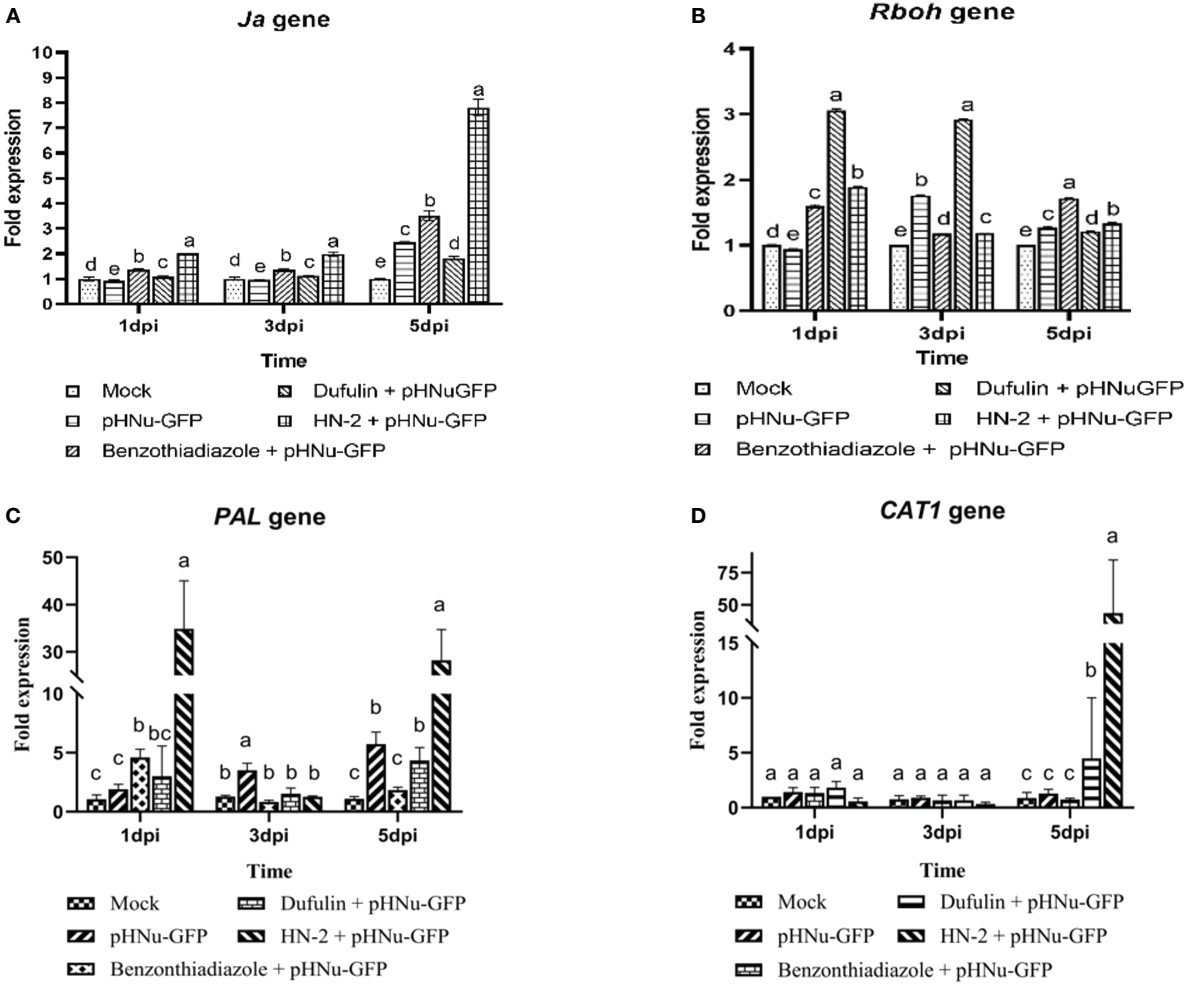
Expression of defense-related genes and jasmonic acid *(Ja)* in *N. benthamiana.* PVMV-GFP in the inoculated leaves of *N. benthamiana* at 1, 3, and 5 dpi at the same time—that is, cultures were incubated for 3, 5, and 7 dpi. **(A)** qRT-PCR analysis showing the expression profile of *Ja* in the inoculated leaves of *N. benthamiana* and treated in sterile water, pHNu-GFP, benzothiadiazole, dufulin, and HN-2, and expression levels were represented as a fold change and normalized to the actin gene. Effects of *B*. *velezensis* HN-2 on the expression of defense-related enzyme genes in the leaves of *N. benthamiana*. Tissue samples were collected for qRT-PCR analysis and the expression of *N. benthamiana* in sterile water (mock), pHNu-GFP, benzothiadiazole-, dufulin-, and HN-2-pHNu-GFP treatments at 1, 3, and 5 dpi (that is, cultures were incubated for 3, 5, and 7 dpi). Expression levels (means ± SD) were represented as a fold change and normalized to the actin gene. **(B)** Respiratory burst oxidase homolog (*Rboh*), **(C)** phenylalanine ammonia-lyase (*PAL*), and **(D)** catalase1 (*CAT1*). The experiment was repeated three times, and the data were normalized according to the 2^-△△CT^ method. Different letters indicate statistically significant differences between treatments according to Duncan’s multiple-range test at *P* < 0.05. Values represent means standard deviations (SDs) from three independent experiments; different lowercase letters indicate a highly significant difference (P < 0.05).

To further validate the mechanism underlying resistance after HN-2 treatment, we quantified the expression of jasmonic acid (JA), resistance-related genes (*Rboh*, *PAL*, and *Cat1* gene) and PR protein genes (*NPR1*, *PR-1b*, *PR3*, and *PR5* gene) in *N. benthamiana* using RT-PCR. We investigated the viral suppression mechanism in response to HN-2 treatment by examining the expression of *JA*, a key regulatory factor of induced systemic resistance (ISR) (Zhou and Wang, 2018). As depicted in **Figure 5A**, *JA* expression gradually increased from 1 to 3 dpi and peaked at 5 dpi within HN-2-treated plants. Notably, the fold change was significantly higher compared to the blank control samples as well as BTH and dufulin treatments with values of 7.81-, 2.22-, and 4.29-fold, respectively.

Respiratory burst oxidase homology (*Rboh*)-mediated H_2_O_2_ generation plays a crucial role in plant growth, development, and response to adverse environmental conditions (Chen and Yang, 2020). In the current study, we observed a significant increase in the expression of Rboh in dufulin-treated plants at 1 and 3 dpi, while BTH treatment exhibited the highest expression among all treatments at 5 dpi. However, compared to the blank control, HN-2 treatment resulted in a modest increase of *Rboh* expression by 1.89-, 1.18-, and 1.34-fold at 1, 3, and 5 dpi, respectively, albeit lower than that of the positive controls (**Figure 5B**). Phenylalanine ammonia-lyase (*PAL*) is a key regulator enzyme involved in the phenylpropanoid pathway for polyphenolic compound production. In comparison to mock-inoculated plants in the present study, the expression of *PAL* was significantly upregulated in HN-2-treated leaves, with the relative expression levels peaking at 24 h and reaching a level 32.61-fold higher than the blank control before returning to near-normal levels within 72 h. Subsequently, there was an increase by 25.58-, 15.33-, and 6.47-fold compared with BTH- and dufulin-treated controls at 5 dpi, respectively (**Figure 5C**). **Figure 5D** demonstrated a similar expression of *Cat1* across all treated plants at 1 and 3 dpi. Furthermore, *Cat1* expression was markedly increased in HN-2-treated leaves, reaching its peak level at 5 dpi with 49.84-, 58.27-, and 9.69-fold higher expressions than those observed for blank control, BTH-treated plants, and Dufulin-treated plants, respectively.

**Figure 6:**
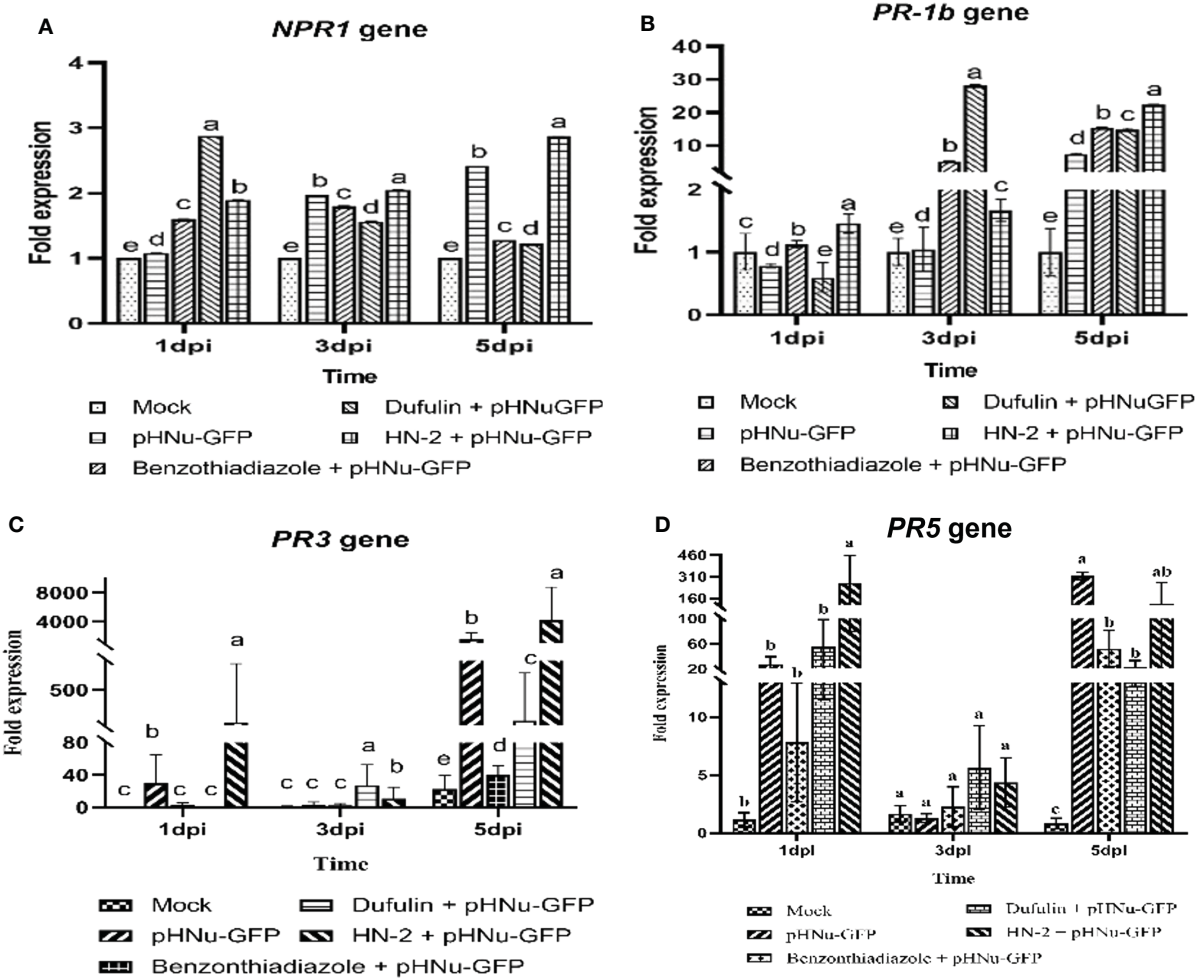
Expression of pathogenesis-related protein genes of *N. benthamiana*. qRT-PCR analysis showing the transcript levels (means ± SD) of PR protein genes in sterile water (mock), pHNu-GFP, benzothiadiazole-, dufulin-, and HN-2-pHNu-GFP treatments of *N. benthamiana* at 1, 3, and 5 dpi (that is, cultures were incubated for 3, 5, and 7 dpi). The expression levels were represented as a fold change and normalized to the actin gene. **(A)** Nonexpressor of pathogenesis-related genes 1 (*NPR1*), **(B)** pathogenesis-related proteins 1b (*PR1b*), **(C)** pathogenesis-related proteins 3 (*PR3*), and **(D)** pathogenesis-related proteins 5 (*PR5*). The experiment was repeated three times, and the data were normalized according to the 2^-△△CT^ method. Values represent means standard deviations (SDs) from three independent experiments; different lowercase letters indicate a highly significant difference (P < 0.05).

Following viral infection, the expression levels of *NPR1*, *PR-1b*, *PR3*, and *PR5* genes gradually increased over time in the leaves of *N. benthamiana* treated with HN-2. We quantitatively analyzed the gene expression at 1, 3, and 5 dpi. In the present study, *NPR1* and *PR-1b* exhibited a gradual increase in the HN-2-treated plants and peaked at 5 dpi. The expression of *NPR1* gene was found to be significantly higher (2.87-, 2.24-, and 2.35-fold) compared to the blank control as well as BTH-treated and dufulin-treated plants (**Figure 6A**). Similarly, the expression levels of the *PR-1b* gene showed significant increases of 22.26-, 1.45-, and 1.50-fold compared to the blank control, BTH-treated plants, and dufulin-treated plants (**Figure 6B**). Interestingly, the result showed that the expression levels of *PR3* and *PR5* genes were significantly upregulated at 1 dpi, followed by a subsequent decrease and eventual peak at 5 dpi. At both time points (1 and 5 dpi), there was a substantial increase in *PR3* gene expression in HN-2-treated plants compared to all three controls: blank control (287.00-fold), BTH-treated (95.32-fold), and dufulin-treated (281.00-fold), respectively (**Figure 6C**). Similarly for *PR5* gene, the expression levels remained significantly higher in HN-2-treated plants than the blank control and BTH- and dufulin-treated plants with a respective fold increase of 312.44-, 45.52-, and 31.88-fold, respectively, at 1 dpi and 285.14-, 5.40-, and 12.30-fold increase at 5 dpi (**Figure 6D**). These findings indicate that HN-2 treatment substantially enhanced PR gene expression in PVMV-infected *N. benthamiana*.

### 3.5 Effect of colonization in *C. chinense* by *B. velezensis* strain HN-2

**Figure 7:**
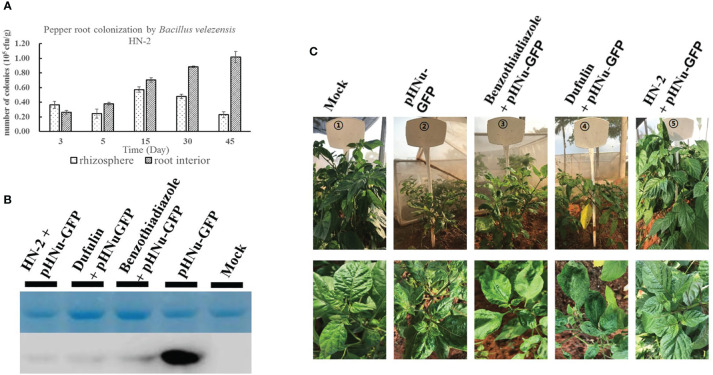
Colonization of *B*. *velezensis* HN-2 in *Capsicum chinense*. **(A)** *B. velezensis* HN-2 colonization experiment in the rhizosphere and root interior of *C*. *chinense* against PVMV infection. The 3, 5, 15, 30, and 45 dpi post-inoculation were set for observation. **(B)** The accumulation of pHNu-GFP protein was detected by western blotting at 60 dpi. **(C)** The strain HN-2 (OD600 = 0.9) was irrigated in the soil of *C*. *chinense*. Benzothiadiazole, dufulin, and sterile water were sprayed on the leaves of *C. chinens*e 48 h after spraying or irrigation, and then these were inoculated with PVMV. The infection of PVMV-GFP in *C*. *chinense* was observed at 180 dpi. All experiments were repeated three times with three independent plants per time.

One-month generated *C. chinense* seedlings were then cultivated in nutrition potting soil mixed with sterile water containing 10^6^ CFU mL^-1^ of *B. velezensis* strain HN-2-GFP under controlled greenhouse conditions to confirm the number of colonies in *C. chinense* rhizosphere soil and root interior soil at 3, 5, 15, 30, and 45 dpi. Our results showed that rhizosphere soil colonization initially increased and then decreased over time, reaching its highest level at 15 dpi before gradually declining at both 30 and 45 dpi. In contrast, root colonization was lower than rhizosphere colonization at the early stage (3 dpi), but it gradually increased with prolonged treatment duration and surpassed rhizosphere colonization by 5 dpi. Subsequently, root interior soil exhibited a high level of colonization at 45 dpi (**Figure 7A**).

### 3.6 Effect of *C. chinense* by *B. velezensis* strain HN-2 on resistance to PVMV in the field

To evaluate the PVMV resistance function of *B. velezensis* HN-2 under field conditions, we subjected field-grown *C. chinense* plants to irrigation with strain HN-2 in soil. Additionally, the leaves of *C. chinense* were sprayed with benzothiadiazole, dufulin, and sterile water, respectively. At 2 days after spraying or irrigation, the plants were inoculated with PVMV. At 60 and 180 dpi after planting, leaf samples were collected, and quantitative analysis was performed for five treatments: sterile water, pHNu-GFP, benzothiadiazole + pHNu-GFP, dufulin + pHNu-GFP, and HN-2 + pHNu-GFP. Western blotting was used to detect the accumulation of PVMV-GFP protein in 60 dpi after inoculation. The results showed that high levels of GFP protein were only detected in pHNu-GFP treatment. GFP protein was almost undetectable in plants treated with benzothiadiazole, dufulin, and *B. velezensis* HN-2 (**Figure 7B**). We observed that the growth of *C. chinense* treated with HN-2 exhibited a significant improvement compared to those treated with pHNu-GFP as well as BTH and dufulin treatments, similar to the blank control at 180 dpi (**Figure 7C**). According to a statistical analysis of morbidity rates, no morbidity was observed in the group receiving only water treatment, almost 100% incidence rate was observed in the group receiving only PVMV (pHNu-GFP), and incidences for BTH and dufulin treatments were at 80% and 75%, respectively, whereas the HN-2 treatment group showed a significantly lower incidence rate of only 60%.

## 4 Discussion

PVMV stands as a prominent plant viral ailment in Hainan province’s fields, causing substantial economic losses since its recent emergence. Limited effective pesticides and chemical treatments exist to combat PVMV infection. Previous studies have shown that *Bacillus* spp. can promote plant growth, and its metabolites can significantly inhibit the effects of plant pathogens, which are commonly used in plant disease management (Fira et al., 2018; Abdelkhalek et al., 2020). These metabolites offer a wide range of secondary compounds that may stimulate plant ISR while impeding pathogen growth (Fira et al., 2018; Vurukonda et al., 2018). Although ISR using *Bacillus* has shown promise against various plant viruses, such as TMV, CMV, and PVY (Murphy et al., 2000, 2003; Wang et al., 2009), its efficacy against PVMV remains unexplored. In this study, we assessed the antiviral properties of *Bacillus velezensis* HN-2 and its chemical counterparts. Our study revealed that treatment with *B. velezensis* strain HN-2 reduced the PVMV infection rates, symptoms, and disease severity in *Capsicum chinense*, correlating with reduced virus quantities in the leaves. These findings underscore the potent control efficacy of *B. velezensis* HN-2 against PVMV infection in *C. chinense*, further affirming its role as a plant growth-promoting rhizobacterium capable of inducing plant-induced systemic resistance against PVMV by eliciting JA production.

Benzothiadiazole and dufulin are exogenous chemical small molecule plant disease activators that lack antimicrobial activity *in vitro* but induce the accumulation of reactive oxygen and phenolics, enhance defense enzyme activity, induce disease-course-related protein expression in plants, activate the SA signaling pathway, and prompt SAR antiviral immune mechanisms to improve the inhibition of viral infection (Chen et al., 2012; Song et al., 2014; Zhou and Wang, 2018; Huang et al., 2020; Wang et al., 2021). While chemical elicitors effectively manage viral diseases, they often result in significant growth penalties in many cases (Heil and Baldwin, 2002). Previous studies by Azami-Sardooei et al. (2013) observed no changes in plant size or yield of tomatoes treated with BTH relative to that in the controls, except for low-level leaf stunting and slight leaf scorching at high concentrations (1,000 mg L^-1^). However, in cucumbers and beans, 100 mg L^-1^ BTH treatment reduced the plant size, growth rate, and flower and fruit numbers. Spychalski et al. (2021) demonstrated that BTH-mediated induction of SAR may alter plant resource allocation, leading to a growth–immunity trade-off and potentially reducing yield. Furthermore, Yu et al. (2021) found that dufulin exposure affected the fatty acid transport involved in carnitine formation, altering free fatty acid concentrations and Tubifex’s oxidative damage response. Moreover, dufulin may act on the urea cycle by inhibiting ASL, causing urea cycle disorder. However, the long-term toxic effects of BTH and dufulin on plants and the environment remain unclear. Conversely, *B. velezensis* is recognized as an important biocontrol strain capable of triggering disease resistance across various plant species while being environment friendly, as it completely degrades in soil without residue (Ye et al., 2018).

Recent studies have emphasized that *B. velezensis* can induce plant disease resistance through various cluster genes involved in the synthesis of non-ribosomal and ribosomal secondary metabolites, volatile compounds, and cyclic lipopeptides with antimicrobial properties; these genes also act as stimulators of ISR (Ye et al., 2018; Borriss et al., 2019). A previous study revealed the potent inhibitory effect of *B. velezensis* strain PEA1 against *F. oxysporum* and CMV infections, suggesting its potential as a biocontrol agent (Abdelkhalek et al., 2020). Vinodkumar et al. (2018) reported the effective reduction of TSV incidence by *B. amyloliquefaciens* (VB7) under field and glasshouse conditions. Similarly, Lee and Ryu (2016) observed no reduction in fruit yield with *Bacillus amyloliquefaciens* strain 5B6 treatment compared to that in water-treated control plants. However, BTH treatment consistently reduced fruit yield in pepper. Moreover, few studies have directly compared the efficacy of biological controls and chemical inducers of plant immunity (such as dufulin and BTH) against plant viruses. In the present study, we selected BTH and dufulin as positive controls and revealed that the primary mechanism of *B. velezensis* HN-2 resistance to PVMV is the induction of systemic resistance. Under greenhouse conditions, soil and plant root irrigation with *B. velezensis* strain HN-2-GFP, combined with foliar application of benzothiadiazole and dufulin 48 h before viral inoculation, significantly (*P* < 0.05) reduced the disease symptoms and PVMV accumulation levels in treated tobacco plants compared to those in the PVMV-, BTH-, or dufulin-treated plants at 7 dpi. Visualization of green fluorescent signals in the inoculated leaves treated with *B. velezensis* HN-2 revealed much lower intensities at 5 and 7 dpi compared to those in negative and positive controls. This reduction was accompanied by mosaic patterns and severe shrivel symptoms in the negative and positive controls, while no evident symptoms were observed in the mock or *B. velezensis* HN-2 extract-treated tobacco plants.

In DAB staining, 3,3-diaminobenzidine was used to visually detect H_2_O_2_, as ROS generation is often associated with plant cell death (Zhu et al., 2013). Oxidized DAB precipitates as a brown color at the peroxidase site and is observable via light microscopy (Hans et al., 1997; Liu et al., 2014; Zhu et al., 2016). Furthermore, with increasing processing time, the leaves of plants treated with PVMV, BTH, and dufulin gradually darkened. By the 7th day, the PVMV-treated leaves exhibited substantial browning, whereas those treated with BTH and dufulin displayed dark brown precipitates covering approximately two-thirds of the leaf area. Conversely, HN-2-treated leaves showed the least browning compared to the negative and positive controls, with water-treated leaves appearing almost transparent. These DAB staining results were consistent with the results of green fluorescence analysis. Western blot analysis of young leaves at 15 dpi also showed similar results. These results strongly suggest that *B. velezensis* HN-2 not only exerts a strong antiviral effect but also exhibits sustained efficacy. This may be attributed to the *B. velezensis* HN-2-mediated induction of plant ISR, conferring robust resilience in *N. benthamiana* against PVMV-GFP infection and sustaining its antiviral effects over time.

Plants exhibit specific resistance mechanisms when faced with adverse conditions. HR refers to programmed cell death triggered by an exaggerated response at the infection site. Various reports have indicated that HR causes rapid death of plant cells, which can effectively limit pathogen growth at the infection site, thereby preventing further spread to the surrounding healthy tissues and activating plant resistance responses (Guo et al., 2020; Wang et al., 2021). Viral infections can induce a burst of ROS in host plants, resulting in oxidative stress (Yang et al., 2018). Notably, H_2_O_2_, a type of ROS, serves as an early mediator of plant resistance (Wu et al., 2015). Accordingly, the rapid generation of ROS is a preliminary process by which plants respond to pathogen challenges, with H_2_O_2_ being capable of initiating cell death during the HR response (Delledonne et al., 2001). Plant cells defend themselves against the oxidative damage caused by ROS through the production of antioxidant enzymes, such as SOD, POD, CAT, MDA, and PAL, which play important roles in plant defense against pathogens and counteract viral infection (Zhang et al., 2016; Lv et al., 2020). Guo et al. (2019) found that following viral infection, POD, PPO, and PAL activities increased more rapidly in the leaves of Ba13-treated plants than in those of the controls. The antiviral activity of *Bacillus velezensis* PEA1 induces systemic resistance to CMV, with significantly elevated transcriptional levels of *PAL*, *CHS*, *HQT, PR-1*, and *POD* (Abdelkhalek et al., 2020). Lv et al. (2020) reported that an increase in SOD, POD, CAT, and MDA activities leads to the activation of the plant immune system to combat TMV infection. Similarly, our study revealed that HN-2 treatment reduced PVMV-induced oxidative damage by increasing SOD, POD, and CAT activities compared to those in the blank and negative controls, thereby achieving antiviral effects. These results suggest that SOD, POD, and CAT eliminate excess peroxides, thereby preventing oxidative damage. In contrast, the activity of MDA after HN-2 treatment was lower than that of the blank and negative controls, with no significant difference in activity observed from 1 to 5 dpi following pHNu-GFP inoculation. Following stress exposure, the upregulation of CAT can minimize oxidative stress, which plays a critical role in preventing oxidative damage and protecting plant cells from the oxidative damage caused by ROS (Blackman and Hardham, 2008; Abdelkhalek et al., 2021). In our experiments, the expression of *Cat1* remained consistent across all treatments at 1 and 3 dpi, with rapid accumulation observed in the HN-2-treated plants at 5 dpi, aligning with changes in CAT enzyme activities, demonstrating the efficacy of *B. velezensis* HN-2 in protecting cells from ROS-induced damage, and inducing long-lasting systemic resistance in *N. benthamiana* following primary inoculation.

SAR and ISR are two major forms of plant defense, wherein the plant hormones SA, JA, ABA, and ET play key roles in regulating the signaling networks associated with plant defense against pathogens. SA is essential for SAR activation in tissues distal to the infection site, whereas jasmonate and ethylene are required for ISR (Zhu et al., 2016). Abdelkhalek et al. (2020) demonstrated that SAR induced *B. velezensis* PEA1 against CMV-activated gene expression and enzyme activity related to systemic resistance while inhibiting infection. ISR is recognized as an effective biological control method for inducing plant defense against a broad range of pathogens (Nie et al., 2017). JA serves as a regulatory factor of ISR (Zhou and Wang, 2018); previous studies have shown that *B. amyloliquefaciens* Ba13 can enhance plant resistance against TYLCV disease by directly inducing systemic resistance and increasing the number of beneficial microbes in the rhizosphere (Guo et al., 2019). In the present study, the simultaneous determination of JA and SA revealed that the SA content under the HN-2 treatment gradually increased, reaching a peak at 3 dpi, and subsequently decreased. At 5 dpi, the blank control and BTH-treated groups had 1.33- and 1.37-folds higher SA contents than the HN-2-treated group, respectively. Meanwhile, the expression of JA rapidly increased after 3 dpi and peaked at 5 dpi in the HN-2-treated plants; it was 7.81-, 2.22-, and 4.29-folds higher than that in the blank control and BTH- and dufulin-treated groups, respectively. These results indicate that *B. velezensis* HN-2 treatment significantly elevated JA expression, potentially activating resistance-related gene expression and defense enzyme activity to enhance plant-induced systemic resistance, thereby aiding in the control of PVMV infection.

Rbohs are key enzymes responsible for ROS production in response to hormonal and environmental signals in plants and play crucial roles in plant growth, development, and stress responses (Suzuki et al., 2011; Sun et al., 2015). ROS levels are elevated by Rboh enzymes in response to multiple biotic and abiotic stresses (He et al., 2017; Liu et al., 2017; Zhai et al., 2018; Chapman et al., 2019). In the present study, the expression of *Rboh* under the HN-2 treatment increased by 1.89-, 1.18-, and 1.34-folds at 1, 3, and 5 dpi, respectively, compared to that in the blank control group but remained lower than that in the BTH- and dufulin-treated groups. These results suggest that *B. velezensis* strain HN-2 may potentially maintain *Rboh* in a low-expression state to restrict ROS production and consequently reduce PVMV-induced oxidative damage. *PAL* is a key regulatory enzyme of the phenylpropanoid pathway and is involved in the production of polyphenolic compounds (Guo et al., 2020). POD and *PAL* play important roles in plant defenses against pathogens. Wang et al. (2012) observed that tobacco plants with excessive expression of the *PAL* enzyme exhibited strong resistance to TMV infection, whereas plants with inhibited enzyme activity were more susceptible to TMV. Interestingly, in the present study, *PAL* expression was significantly increased under the HN-2 treatment, peaking at 24 h before reducing to near-normal levels at 3 dpi, and subsequently increased by 25.58-, 15.33-, and 6.47-fold compared with BTH- and dufulin-treated controls at 5 dpi, respectively. Conversely, POD activity was lowest at 1 dpi and increased rapidly at 3 dpi, which indicated that *PAL* expression was induced in advance following virus infection and continued to be induced at 5 dpi, thereby enabling sustained POD activity after 3 dpi. At 5 dpi, the HN-2 treatment demonstrated the highest level at 1,660.31 unit mg^-1^ FW, indicating 1.15-, 1.16-, and 1.57-fold increase in activity compared to the BTH treatment, dufulin treatment, and blank control, respectively. This increase in POD activity may activate the plant immune system to combat PVMV infection.

PR proteins are produced in plants during pathogen attacks and constitute vital components of the plant defense mechanism (Sels et al., 2008). Numerous studies have shown that PGPRs boost plant health by improving defense against various pathogens, often associated with JA and ET pathway induction (Beneduzi et al., 2012; Pieterse et al., 2014; Rahman et al., 2015). In tobacco, ISR triggered by non-pathogen PGPR strains is accompanied by the upregulation of PR genes encoding pathogenesis-related proteins, as evidenced by RT-PCR analysis results indicating an increased expression of PR genes upon treatment with *B. velezensis* (Wang et al., 2009). SAR, demonstrated across many plant species, involves the accumulation of PR proteins (*PR-1*, *PR-2*, and *PR-5*) and SA as its molecular basis (Gao et al., 2014). Niu et al. (2011) found that ISR simultaneously activates the SA- and JA/ET-dependent signaling pathways, leading to the induced expressions of *PR1*, *PR2*, *PR5*, and *PDF1.2*. *NPR1* acts as a regulator of SAR and ISR, mediating the SA–JA crosstalk (Pieterse et al., 2014). The *NPR1* (nonexpresser of PR genes) protein serves as a key regulator in transmitting SA and JA/ethylene signals, thus triggering acquired resistance responses (Spoel et al., 2003). Previous studies suggest that the induction of *PR1* gene synthesis may be associated with increased *NPR1* gene expression and conformational changes in the *NPR1* protein (Mou et al., 2003). Nie et al. (2017) showed that root-drench application of *Bacillus cereus* AR156 significantly reduced disease incidence by activating ISR through a mechanism dependent on *NPR1*, leading to the expression of downstream defense-related genes, such as *PR1*, *PR2*, *PR5*, and *PDF1.2*, and activation of cellular defense responses. Similarly, inoculation with *B. amyloliquefaciens* Ba13 improved the defense ability of tomato plants against TYLCV infection by activating the expression of genes and enzymes related to systemic resistance in tomato—for example, by increasing the expression of the resistance-related genes *PR1*, *PR2*, and *PR3* (Guo et al., 2019). The expression of *PR-1b*, *PR3*, *PR5*, and *NPR1* gradually increased over time in the leaves of HN-2-treated *N. benthamiana* after PVMV inoculation; we quantitatively analyzed the gene expression at 1, 3, and 5 dpi. Consistent with previous findings, *B. velezensis* HN-2 treatment significantly increased the transcriptional levels of *NPR1*, *PR-1b*, *PR3*, and *PR5*, with *PR3* and *PR5* showing the most significant upregulation compared to those in the blank control. Our experimental results indicated that *B. velezensis* HN-2 treatment significantly increased PR gene expression and activated plant-induced systemic resistance, thus highlighting the antiviral properties of this strain. From an agronomic perspective, the ISR triggered by PGPR is interesting, given its long-lasting and broad-spectrum protection without growth costs and its minimal potential to promote pathogen resistance (Köhl et al., 2019). In a previous study, *Arabidopsis* plants were root-drenched with *Bacillus cereus* AR156 at 5×10^8^ CFU mL^-1^, with 10 inoculated plants for each genotype. The results showed that AR156 only colonized the roots and significantly reduced disease incidence through the activation of ISR (Nie et al., 2017). Guo et al. (2019) watered tomato plants with a *Bacillus amyloliquefaciens* Ba13 suspension at 10^6^ CFU mL^-1^; after 3 weeks, post-bacterial inoculation performed viral infection, wherein 65 replicates were sampled for systemic resistance-related assays and virus quantification. It was found that the number and distribution of rhizosphere-dominant bacteria were changed by Ba13 application and can enhance plant resistance against TYLCV disease through the direct induction of systemic resistance. To elucidate the relationship between the colonization of *B. velezensis* HN-2 in *C. chinense* roots and its role in plant virus resistance, a total of 600 plants were subjected to root drenches with *B. velezensis* strain HN-2 at 10^6^ CFU g^-1^ under greenhouse cultivation conditions. The results demonstrated that *B. velezensis* HN-2 rapidly colonized the rhizosphere within 72 h, and with the extension of treatment time, HN-2 mainly colonized the root interior after 5 dpi. Subsequently, the number of colonies in *C. chinense* root interior increased by fivefold compared to that in the rhizosphere. Western blotting of PVMV-GFP protein accumulation after 60 days of inoculation showed that high levels of GFP protein were detected only in pHNu-GFP treatment, while when treated with benzothiadiazole, dufulin, and *B. velezensis* HN-2, GFP protein was almost undetectable in plants. Simultaneously, at 60 days after PVMV-GFP inoculation, a comparative experiment was conducted on the antiviral effect of *B. velezensis* HN-2 on *C. chinense* plants grown in soil that was irrigated under field conditions. We observed that the growth of *C. chinense* treated with HN-2 was significantly superior to that of PVMV-infected plants treated with BTH and dufulin, resembling that of the blank control at 180 dpi in the field.

**Figure 8:**
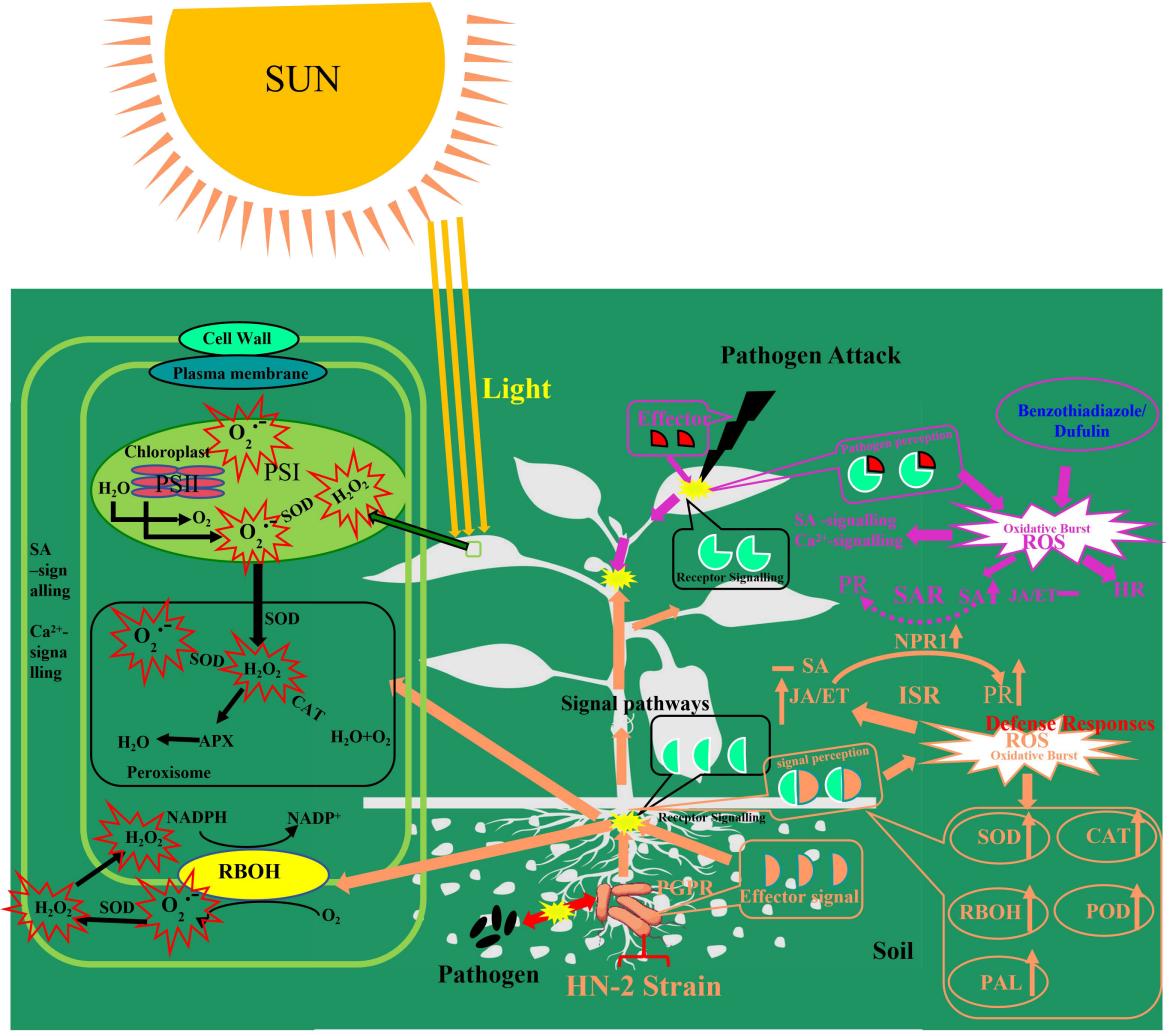
Conceptual model depicting the mechanisms illustrating how symbiosis and convocation probiotics by *B*. *velezensis* on the rhizosphere and soil ultimately inhibit plant pathogens (plant disease and virus).

In a word, the data presented in this study indicate that *B. velezensis* HN-2 functions as a PGPR, rapidly colonizing *C. chinense* roots and activating the expression of genes and enzymes associated with systemic resistance. When plants were infected with PVMV and treated with *B. velezensis* HN-2, the JA signaling pathway was activated, leading to increased *JA* content and the simultaneous activation of the *NPR1*-dependent mechanism, resulting in the significant upregulation of *PR-1b*, *PR3*, and *PR5*. Moreover, cellular defense responses, including CAT, POD, *Rboh*, *PAL*, and *CAT1* activities, exhibited a more rapid and significant increase. Furthermore, the increased expression of JA pathway genes suggests the activation of ISR under *Bacillus velezensis* HN-2 treatment. However, there was no significant increase in SA content, indicating that systemic acquired resistance (SAR) pathway was not induced by this treatment (**Figure 8**). In conclusion, *B. velezensis* HN-2 can effectively induce plant ISR-mediated defense responses and enhances plants’ immunity (including *C. chinense*) by activating the JA signaling pathway, thereby mitigating symptoms caused by PVMV and safeguarding the plants against pathogenic infections.

## 5 Conclusions

In summary, *Bacillus velezensis* HN-2 is a new antiviral agent against plant viruses and plays significant roles in ISR by activating gene expression and enzyme activity related to systemic resistance, thus inhibiting infection. The strain HN-2 may be considered a promising source of plant growth promotion, with highly effective antiviral substances for plant protection and for development against PVMV diseases. This study provides theoretical basis for the green prevention and control of plant virus diseases.

## Data availability statement

The datasets presented in this study can be found in online repositories. The names of the repository/repositories and accession number(s) can be found in the article/**Supplementary Material**.

## Author contributions

PJ: Formal analysis, Funding acquisition, Supervision, Writing – review & editing. ZX: Data curation, Investigation, Writing – original draft. YW: Data curation, Investigation, Writing – original draft. YS: Data curation, Methodology, Writing – original draft. XP: Data curation, Investigation, Writing – original draft. JW: Data curation, Investigation, Writing – original draft. WL: Formal analysis, Resources, Writing – review & editing. WM: Supervision, Writing – review & editing.
