# Supplementary material for: Bacillus velezensis HN-2: a potent antiviral agent against pepper veinal mottle virus
Source: Front Plant Sci. 2024 Jul 10;15:1403202. doi: 10.3389/fpls.2024.1403202 (PMC11266135; doi:10.3389/fpls.2024.1403202)
Supplement: Supplementary file 1 [file Table_1.docx]

Supplementary Material

# Supplementary Table

**Supplementary Table 1 Primers for Specific qRTPCR**

| Primers | Sequence （5'—3'） |
| --- | --- |
| ActinF | TTCAAGGGTGCTTCAGTA |
| ActinR | TTATGGTTGGTATGGGTC |
| CAT1F | CCAATAAGCTACCACTACGAAACG |
| CAT1R | GGGAAAGAGGAGTGAACCCATT |
| JAR | ACGGCTACCTTGTTACGACTT |
| JAF | AGAGTTTGATCCTGGCTCAG |
| *NPR1*F | GCAGCAGACGATGTAATG |
| *NPR1*R | GCAGCAGACGATGTAATG |
| PALF | CAGCCCAAGGAAGGATTG |
| PALR | TCAGAAAGGACAGCAAGAATGT |
| *PR1b*F | GATGCCCATAACACAGCTCGTGC |
| *PR1b*R | GCCTCTATAATTACCTGGAGGATC |
| *PR3*F | CCAGAGTGACAGATATTA |
| *PR3*R | GCCCTGGCCGAAGTTCCT |
| *PR5*F | GTCAACCAATGCACCTAC |
| *PR5*R | GGTGGATCATCCTGTGGA |
| *RBOH*F | CTTGCTCGTCAACATCGTG |
| *RBOH*R | GGAGAAATCTTGTTGAGAGC |
